# Editorial: Genetics and genomics of emerging and multifactorial stresses affecting plant survival and associated plant microbiomes

**DOI:** 10.3389/fpls.2025.1738816

**Published:** 2025-12-17

**Authors:** Somashekhar M. Punnuri, Mahendar Thudi, Reyazul Rouf Mir

**Affiliations:** 1 Agriculture Research Station, College of Agriculture Family Science and Technology (CAFST), Fort Valley State University, Fort Valley, GA, United States; 2 Department of Genetics and Plant Breeding, Sher-e-Kashmir University of Agricultural Sciences & Technology of Kashmir (SKUAST-K), Srinagar, Kashmir, India

**Keywords:** genome-wide association studies (GWAS), Transcriptomics, QTL mapping, epigenetic profiling, functional genomics, microbiome research, stress resilience, holobiont

In the face of unprecedented global climate challenges, agricultural systems must adapt to a convergence of multifactorial stresses—drought, salinity, temperature fluctuations, nutrient limitations, pathogen outbreaks and other abiotic stresses—many of which occur concurrently, amplifying their impact and threatening crop yield, quality, and global food security (Fedoroff et al., 2010; Jiang et al., 2025). These multifactorial stressors not only threaten global crop yields and food security but also expose the underexplored but critical role of plant-associated microbiomes in mediating plant health, development, and resilience. This complex biological interplay provides the backdrop for the Research Topic *“Genetics and Genomics of Emerging and Multifactorial Stresses Affecting Plant Survival and Associated Plant Microbiomes”.*

Bringing together sixteen original research and one mini-review articles, this Research Topic presents a multifaceted exploration into how plants perceive, respond to, and adapt under layered stress conditions. Through the integration of genome-wide analyses such as Genome-Wide Association Studies (GWAS), transcriptomics, QTL mapping, epigenetic profiling, functional genomics and microbiome research, these studies not only deepen our understanding of plant stress biology but also lay the groundwork for a paradigm shift in crop improvement—one that embraces holobiont-based breeding (Huitzil et al., 2023) and systems-level thinking for resilience in a rapidly changing climate.

## Advancing genetic dissection through genetic mapping, GWAS and QTL meta-analysis

Several contributions leverage powerful genomics tools to identify genes and markers associated with stress-related traits. Vutla et al. dissected the genetic basis of eight key traits in pearl millet (*Cenchrus americanus*) using a recombinant inbred line population and high-density SNP map, identifying 45 QTLs. The co-localization of multiple QTLs on LG3 and consistent detection across years emphasizes the robustness and breeding relevance for the yield-related traits and also suggesting linked improvement of traits like plant height and panicle size in pearl millet. The overlap of QTLs means breeders can potentially improve multiple traits with fewer selection cycles. This work helps secure millet’s role as a hardy cereal for food and fodder in marginal lands. Sahu et al. conducted a comprehensive meta-analysis consolidating QTL data from 30 studies over 12 years, leading to the identification of 70 high-confidence meta-QTLs associated with yield, stress tolerance, and aflatoxin resistance in peanut (*Arachis hypogaea*). The discovery of candidate genes linked to aflatoxin resistance and fatty acid composition offers direct targets for marker-assisted programs. This sets the stage for peanuts that are safer, healthier, and more climate-resilient. Chandana et al. conducted a large-scale genome-wide association studies (GWAS) in chickpea (*Cicer arietinum*), identifying over 1,000 marker–trait associations, including 75 novel loci related to root nodulation. Their work provides critical genetic insights into enhancing biological nitrogen fixation, an essential trait for sustainable crop production. In wheat, Sharma et al. employed QTL meta-analysis to consolidate over 200 QTLs associated with powdery mildew resistance into 68 meta-QTLs and 13 high-confidence MQTLs, some co-localizing with known resistance genes in wheat. The refined loci enable fine mapping and functional studies to enhance durable resistance. Complementarily, Vishwakarma et al. performed GWAS to map SNPs linked to grain quality and agronomic traits in bread wheat (*Triticum aestivum*). Their identification of stable, environment-resilient SNPs informs breeding programs aimed for dual improvement in yield and quality. Suresh et al. screened 427 tropical maize lines and identified 14 lines with robust Gray Leaf Spot resistance, some carrying extra drought or viral resistance. The genetic markers found provide a toolkit for breeders to combine disease resistance with agronomic performance. Given GLS’s yield impact in Africa, these donor lines could substantially reduce crop losses without heavy fungicide use. Together, these studies illustrate the power of modern genomic tools to untangle complex stress-related traits and move toward the development of crop varieties that are not only high-yielding but also resilient to multifactorial environmental stresses. Their collective contributions mark a critical step in incorporating multi-trait and environment-stable genomic regions into mainstream breeding programs.

## Molecular, epigenetic, and evolutionary insights of gene families in plant defense and disease resistance

Understanding gene expression and regulatory mechanisms under stress is essential for designing resilient crops. Ahmad et al. explored the transcriptomic response of date palm roots to salinity stress in the presence of the beneficial root endophyte *Piriformospora indica*. Their study revealed upregulation of genes involved in ion transport, oxidative stress responses, and hormone signaling highlighting the synergistic roles of beneficial microbes in stress mitigation. Liu et al. conducted a comprehensive metabolomic and microbial profiling of tobacco rhizosphere soils across four cropping systems, using non-targeted metabolomics and amplicon sequencing (16S rRNA and ITS). Their findings revealed significant shifts in lipid metabolism, amino acid biosynthesis, and secondary metabolite pathways, which in turn shaped distinct microbial communities. Notably, increased abundance of arbuscular mycorrhizal and saprotrophic fungi suggests enhanced nutrient cycling and plant support under diversified cropping. This study demonstrates the intricate link between soil chemistry and microbial dynamics and provides actionable insights for designing sustainable rotation and fertilization strategies in tobacco cultivation. Patil and Tripathi profiled microRNA expressions in papaya (*Carica papaya*) genotypes with contrasting responses to Papaya ringspot virus (PRSV), revealing genotype-specific and infection-responsive miRNA signatures. Their findings underscore the regulatory role of small RNAs in modulating antiviral defenses and highlight miRNA-based mechanisms that differentiate susceptible and resistant responses. This study opens avenues for molecular breeding and genome-editing approaches to enhance PRSV resistance in papaya. Su et al. provided a comprehensive analysis of Protein Arginine Methyltransferase (PRMT) and Jumonji C-domain containing (JmjC) gene families in apple (*Pyrus malus*), linking their expression to cold and drought stress. As key players in histone modification and epigenetic regulation, these genes are promising candidates for engineering stress-responsive chromatin dynamics in perennial crops. Adding an evolutionary dimension, Sultan et al. studied NLR immune gene evolution in annual and perennial Glycine species. Annuals like soybeans had expanded NLRomes via recent duplications, while perennials experienced contraction but higher diversification. Gene birth after speciation contributed to unique repertoires, especially in *G. latifolia*. Uneven NLR distribution was observed in polyploid genomes. Findings inform disease resistance breeding in soybean. This study underscores the value of leveraging wild relatives for breeding programs and highlights the broader importance of germplasm diversity in anticipating future pathogen pressures. Evolutionary divergence and gene duplication events have shaped plant immunity-related gene families (e.g., NLRs, PRMTs, JMJs), offering novel genetic resources for biotechnological and breeding interventions.

Collectively, these insights reveal the sophisticated regulatory frameworks plants employ to navigate environmental stress and emphasize untapped avenues for breeding resilient cultivars through the integration of molecular and evolutionary principles.

## Functional genomics and field validation

Several studies bridged molecular discoveries with practical applications. Wambi et al. employed a multi-trait, principal component–based selection index on 192 maize hybrids to identify genotypes resistant to fall armyworm (FAW). The best index improved yield under infestation while cutting leaf damage significantly. This approach lets breeders target multiple traits at once, speeding development of pest-resilient hybrids. Such tools could be crucial for African farmers battling FAW without heavy pesticide reliance. This approach offers a robust and efficient tool for breeding high-yielding, FAW-resistant maize varieties. Kavai et al. investigated the genetic basis of resistance to maize lethal necrosis (MLN) in tropical maize by evaluating 182 hybrids from a 14-parent diallel across three years under artificial inoculation and rainfed conditions in Kenya. Identifying inbred lines with both resistance and yield potential allows for hybrid development without major trade-offs. In regions hit by MLN, these results offer a pathway to stable maize production in sub-Saharan Africa. Wang et al. offer a mechanistic foundation for hemiparasitic seedling development in *Malania oleifera*, an ecologically significant and oil-rich tree endemic to karst regions. Growth trials with nutrient-rich/poor soils and various hosts showed vigorous hosts greatly improved aboveground growth, with less effect on roots. Hormone metabolism, stress response, and antibiotic biosynthesis genes were upregulated in haustoria. Host association boosted nutrient synthesis and stress tolerance. Findings aid cultivation of hard-to-grow hemiparasites while optimizing propagation strategies for this economically valuable species.

## Plant-microbiome interactions under multifactorial stresses

An essential component of this Research Topic is the examination of plant-microbiome interactions in stress contexts: Rhizosphere microbial communities are dynamic mediators of plant stress responses and disease resistance. Crop genotype, soil conditions, and biotic stresses co-influence microbial shifts, with strong implications for sustainable agriculture.

Liu et al. studied tobacco cropping systems, showing that crop rotation and fertilization alter rhizosphere metabolites (lipids, amino acids) and microbial diversity (e.g., mycorrhizae), enhancing soil health and plant productivity. Tyagi et al. provide a timely mini-review on the complex interplay between waterlogging stress, plant microbiomes, and disease development. The authors emphasized how waterlogging induces metabolic reprogramming, hypoxia, nutrient imbalances, and shifts in microbial community structure, all of which can exacerbate pathogen incidence and compromise plant resilience. This synthesis identifies critical knowledge gaps and lays the groundwork for integrating microbial ecology into waterlogging-tolerant crop management strategies under climate change scenarios. Karapareddy et al. profiled rhizosphere microbial communities in cotton-growing soils across North Alabama with varied levels of reniform nematode infestation using 16S and ITS amplicon sequencing. Their study identified over 47,000 bacterial and 3,400 fungal ASVs, with key bacterial genera such as *Bacillus*, *Streptomyces*, and *Conexibacter*, and fungal genera including *Fusarium* and *Cladosporium*. The community structure showed tight clustering among Actinobacteria, Acidobacteria, and Proteobacteria, suggesting functional synergy. These findings underscore the ecological relevance of microbial diversity in modulating plant–nematode interactions and provide a foundation for rhizosphere-targeted strategies in pest and soil health management. Deng et al. characterized rhizosphere bacterial communities in oilseed rape (*Brassica napus*) cultivars with contrasting responses to *Plasmodiophora brassicae* infection, the causal agent of clubroot disease. Using amplicon sequencing and metagenomic functional analysis, the study revealed that resistant and susceptible cultivars exhibited distinct shifts in key nitrogen-cycling bacterial genera—such as *Nitrosomonas*, *Limnobacter*, and *Thiobacillus*—under pathogen stress. Notably, susceptible cultivars displayed enhanced bacterial co-occurrence network complexity and upregulation of nitrification genes, while resistant cultivars favored assimilatory nitrate reduction pathways. These findings emphasize the dynamic role of rhizosphere microbiomes in modulating host-pathogen interactions and provide insight into microbial contributions to disease resistance mechanisms. These studies collectively advance the plant–microbiome integration paradigm—the concept that plant resilience emerges from the co-functioning of plant and microbial genomes.

## Roadmap for future research: systems biology and beyond

### Holobiont paradigm and microbiome-driven resilience

One of the most forward-looking themes in this Research Topic is the plant holobiont concept—the recognition that plant performance is co-determined by its microbiome:

- Tyagi et al. reviewed microbial dysbiosis under waterlogging stress.
- Ahmad et al. showed how P. indica inoculation enhances salt tolerance in date palm.

These studies collectively advocate for “holobiont breeding”, integrating host genetic traits with microbiome function—a paradigm shift from conventional plant-centric approaches to co-optimized plant–microbiome systems.

### Towards systems-level crop improvement: challenges and horizons

The articles in this Research Topic signal a transformation in plant biology—from single-gene studies to systems-level, multidimensional investigations. Key future directions identified include:

- Causal microbiome engineering uses synthetic communities and functional metagenomics.
- Temporal and spatial gene expression resolution through high-resolution time-series and cell-type-specific profiling.
- Pan-genome analysis and structural variant discovery beyond SNP-centric studies.
- CRISPR/dCas9 epigenome editing and synthetic RNA technologies for precision trait modulation.
- AI-driven phenotype prediction and climate-resilient variety design through integration of omics and environmental data.

These trajectories call for transdisciplinary collaboration, uniting plant genetics, epigenomics, microbiology, data science, and field-based agronomy.

The 17 articles published under this Research Topic represent a significant leap forward in our collective understanding of plant stress biology. These studies span a diverse array of plant systems, stress types, and methodological frameworks ranging from GWAS, meta-QTL analyses, transcriptomics, metabolomics, and epigenetics to field validation and microbiome profiling. Together, this Research Topic moves beyond single-stressor frameworks by embracing integrative approaches that capture the interactions among plant genomes, epigenomes, and the associated microbial communities-the plant holobiont. Multiple studies have demonstrated that stress resilience is not solely encoded in the plant genome but is also influenced by dynamic plant–microbiome interactions. For instance, insights from Liu et al. Deng et al.,Karapareddy et al. and Tyagi et al. emphasize the role of rhizosphere and endosphere microbial shifts under stress and highlight beneficial microbes as potential bioenhancers. Simultaneously, genetic and genomic studies by Vutla et al., Sahu et al., Suresh et al. and Chandana et al. offer precise markers and candidate genes for resistance breeding through QTL mapping and GWAS. Several contributions exemplify the shift toward actionable trait discovery (Kavai et al. and Wambi et al.) bridging lab-based genomic insights with field-based validation. Moreover, integrative molecular approaches including miRNA profiling (Patil and Tripathi), transcriptome analysis (Ahmad et al.), and epigenetic regulators (Su et al.) advance our mechanistic understanding of stress perception and response. The evolutionary insights from Sultan et al. add further depth by situating stress responses within the broader context of gene family diversification and adaptation. Collectively, these works signal a notable advancement toward designing crops not merely as standalone genetic entities, but as dynamic systems interacting continuously with their environment and associated biota. A compelling future direction emerging from these studies is the incorporation of microbiome-aware selection and breeding a concept gaining traction as “holobiont breeding” (Huitzil et al., 2023). This requires precise characterization of beneficial microbial consortia, their functions, and the plant traits that facilitate their recruitment and persistence under stress.

The future of plant resilience research must integrate multi-omics with high-throughput phenotyping, environmental modeling, and artificial intelligence. Such convergence will enable the discovery of novel gene networks, predictive trait–microbe associations, and adaptive alleles suited to future climates (Wang et al., 2024). Structural variants and presence/absence variations uncovered through pangenomics and Pan-GWAS offer another layer of stress-relevant variation often missed in SNP-only frameworks (Hu et al., 2024, 2025). Advances in genome editing tools like CRISPR/dCas systems and synthetic biology platforms hold promise for targeted modulation of regulatory networks involved in stress tolerance (Miki et al., 2018). Moreover, future research must prioritize stress combinations and field-level complexity. Multifactorial stress experiments, longitudinal sampling, cell-type specific profiling, and synthetic community reconstitution are necessary to elucidate causal interactions and validate lab findings under real-world agricultural scenarios (Schmitz et al., 2022; Taskiran et al., 2024).

## Conclusion

The convergence of climate variability, pathogen pressure, abiotic stress, and soil degradation poses complex challenges to global agriculture. Addressing these requires an integrated understanding of plant genetics, physiology, and plant–microbe interactions. The Research Topic offers a blueprint for the future of sustainable crop improvement. It calls for systems-level integration of plant genetics, microbial ecology, functional genomics, and field-based validation. The holistic vision presented here anchored in robust data and interdisciplinary collaboration will be pivotal in breeding the next generation of climate-smart, microbiome-optimized, and climate-adapted varieties. We are confident that the insights presented in this Research Topic will inspire continued innovation and strategic research toward global food and environmental security. The studies presented herein converge on a singular message: the path to climate-smart agriculture lies in holobiont-aware breeding, where plant genotypes and their associated microbiomes co-evolve and co-adapt for sustainable productivity.

We extend our deepest appreciation to the authors, reviewers, and editorial teams whose contributions make this thematic issue a robust resource and a visionary roadmap for advancing plant resilience research. The insights and innovations presented here pave the way for a future where crops are not only genetically fortified but ecologically and epigenetically empowered to thrive in the face of multifactorial stresses.
